# Differential Dorsolateral Prefrontal Cortex Proteomic Profiles of Suicide Victims with Mood Disorders

**DOI:** 10.3390/genes11030256

**Published:** 2020-02-27

**Authors:** Alejandra Cabello-Arreola, Ada Man-Choi Ho, Aysegul Ozerdem, Alfredo B. Cuellar-Barboza, Mehmet U. Kucuker, Carrie J. Heppelmann, M. Cristine Charlesworth, Deniz Ceylan, Craig A. Stockmeier, Grazyna Rajkowska, Mark A. Frye, Doo-Sup Choi, Marin Veldic

**Affiliations:** 1 Department of Psychiatry and Psychology, Mayo Clinic, Rochester, MN 55905, USA; 2 Department of Neurosciences, Dokuz Eylul University, Health Sciences Institute, Izmir 35340, Turkey; 3 Department of Psychiatry, Dokuz Eylul University, School of Medicine, Izmir 35220, Turkey; 4 Department of Psychiatry, Universidad Autonoma de Nuevo Leon, Monterrey 64460, Mexico; 5 Proteomics Core, Medical Genome Facility, Mayo Clinic, Rochester, MN 55905, USA; 6 Izmir University of Economics, Faculty of Medicine, Department of Psychiatry, Izmir 35330, Turkey; 7 Department of Psychiatry, Case Western Reserve University, Cleveland, OH 44106, USA; 8 Department of Psychiatry and Human Behavior, University of Mississippi Medical Center, Jackson, MS 39216, USA; 9 Department of Molecular Pharmacology and Experimental Therapeutics, Mayo Clinic College of Medicine and Science, Rochester, MN 55905, USA; 10 Neuroscience Program, Mayo Clinic College of Medicine, Rochester, MN 55905, USA

**Keywords:** KCNQ3, DLPFC, GABA, suicide, proteomics, pathways, mood disorders, brain

## Abstract

Suicide is a major public health concern; nevertheless, its neurobiology remains unknown. An area of interest in suicide research is the dorsolateral prefrontal cortex (DLPFC). We aimed to identify altered proteins and potential biological pathways in the DLPFC of individuals who died by suicide employing mass spectrometry-based untargeted proteomics. Postmortem DLPFC from age-matched male suicide mood disorder cases (*n* = 5) and non-suicide mood disorder cases (*n* = 5) were compared. The proteins that differed between groups at false discovery rate (FDR) adjusted *p*-values (Benjamini–Hochberg–Yekutieli) <0.3 and Log_2_ fold change (FC) >|0.4| were considered statistically significant and were subjected to pathway analysis by Qiagen Ingenuity software. Thirty-three of the 5162 detected proteins showed significantly altered expression levels in the suicide cases and two of them after adjustment for body mass index. The top differentially expressed protein was potassium voltage-gated channel subfamily Q member 3 (KCNQ3) (Log_2_FC = −0.481, *p* = 2.10 × 10^−09^, FDR = 5.93 × 10^−06^), which also showed a trend to downregulation in Western blot (*p* = 0.045, Bonferroni adjusted *p* = 0.090). The most notably enriched pathway was the GABA receptor signaling pathway (*p* < 0.001). Here, we report a reduction trend of KCNQ3 levels in the DLPFC of male suicide victims with mood disorders. Further studies with a larger sample size and equal sex representation are needed.

## 1. Introduction

Suicide is a major public health concern and its rates are steadily increasing [1]. Globally, it is estimated that over 800,000 people die by suicide annually [2]. It has been postulated that suicide is the result of an interaction between state-dependent (environmental) stressors and a trait-like diathesis or susceptibility to suicide [3]. Unfortunately, the identification of clinical predictors is scarce, and no biomarkers have been established; therefore, identifying possible underlying biological mechanisms for this vulnerable population is important. 

The dorsolateral prefrontal cortex (DLPFC) has been an area of special interest in suicide research due to evidence of morphometric changes and neurotransmitter abnormalities. Among the structural finding in the DLPFC are changes in the volume [4,5,6] and in the density of neurons [7]. Some neurotransmitters and proteins that have a functional connection to γ-aminobutyric acid (GABA), glutamate, and serotonin receptors have been pointed out as altered in the prefrontal cortex of individuals who died by suicide [8]. In the search for candidate genes involved in suicide and attempted suicide, previous studies have examined genes implicated in neurotransmitter systems, including the GABAergic, glutamatergic, serotonergic, noradrenergic, and dopaminergic systems [9,10]. However, an understanding of the biological pathways affected by these systems is just starting to be integrated.

Proteomics is the study of the total set of expressed proteins by a cell, tissue or organism at a given time under a determined condition [11]. The proteome also includes the modifications made to proteins, which are subjected to time and distinct requirements, stresses, or other environmental factors [12]. Proteomics studies are highly relevant in complex conditions that involve both genetic and environmental factors, as suicide is theorized. Some of these factors that contribute to suicidality are a genetic predisposition, early-life adversity that can result in stable changes to gene expression, neurotransmitter levels alterations, inflammation in the central nervous system and neuroglia, psychopathology, recent life events, behavioral and emotional traits, among others [13]. Proteomic profiles have been investigated in other complex conditions as mood disorders in an effort to discover biomarkers to facilitate accurate diagnosis and treatment [14]. The significance of proteomic studies lies in the quantitative and dynamic information that they are able to provide by directly profiling protein expression. Furthermore, it is possible to discover underlying disease mechanisms by performing biological pathway analysis to these proteins, where experimental results are mapped to knowledge-based comprising known molecular interactions collected from the literature [15]. 

In this study, we aimed to identify altered proteins and potential biological pathways in the DLPFC of individuals with mood disorders who died by suicide in comparison to those who died due to other causes by employing mass spectrometry-based untargeted proteomics. We hypothesized that the suicide group would present an altered DLPFC proteome compared to the non-suicide group.

## 2. Materials and Methods 

### 2.1. Subjects 

The postmortem DLPFC of five male individuals who died by suicide and five male individuals who died by non-suicide causes were procured. Subjects were matched by age individually, and all of them presented a major mood disorder. Working with the brain tissue sample is a challenging task from many perspectives including recruitment, ethical concerns, technical conditions for storage, and tissue sampling [16]. The current study has been performed on brain tissue samples collected over nearly 20 years, still containing a limited number of cases with death by suicide. In addition to that, due to difficulties in procuring postmortem brain tissues of the suicide phenotype for which a description of each participant has been made available in the Supplementary Materials, we had a limited number of participants.

The Institutional Review Boards of the University of Mississippi Medical Center, Jackson, MS, and the University Hospitals Cleveland Medical Center, Cleveland, OH had approved all the procedures and were in accordance with the Declaration of Helsinki. Informed consent from the legally-defined next-of-kin was obtained for the collection of tissue, medical records, and retrospective interviews; this information is also reported in a previous study [17]. Structured Clinical Interview for DSM-IV Axis I Disorders was administered by a Master-level social worker to knowledgeable informants of the subjects. To determine subjects’ psychopathology, a board-certified clinical psychologist and a board-certified psychiatrist independently reviewed the diagnostic interview scoring notation, the medical examiner’s report, any prior medical records, and a comprehensive narrative that summarized all scores of information about each subject. The social worker, the clinical psychologist, and the psychiatrist reached a consensus on the diagnosis. Cause of death was determined by the medical examiner. Subjects in either group who met DSM-IV criteria for bipolar disorder (BD) or major depressive disorder (MDD) diagnosis were included for this study. Subjects with any neurological disorders were excluded. The presence of psychotropic medications and substances of abuse in blood and urine was determined by the medical examiner’s office.

### 2.2. Tissue Collection

Gray matter tissues of DLPFC (Brodmann area 9) were derived from postmortem brains collected at autopsy at the Cuyahoga County Medical Examiner’s Office, Cleveland, OH. Tissues were dissected and rapidly frozen in 2-methylbutane on dry ice without fixation, and were kept in dry ice during transportation before permanent storage at -80 °C. 

### 2.3. Sample Preparation: Tissue lysis 

Sections of stored frozen DLPFC tissue were cut and transferred to pre-tared tubes for wet weight measurement. A 15× (wt/vol) volume of cold lysis buffer (0.1% SDS/50mM Tris, pH 8.2/1mM MgCl2/Halt Protease Inhibitor (ThermoFisher Scientific, Bremen, Germany)/Simple Step1+3 phosphatase inhibitor (GoldBio)/Benzonase (Invitrogen) was added to 2 mL ceramic bead tubes on ice. Frozen brain tissue was transferred by forceps into the bead tube, and the tissue was homogenized using a Minilys (Bertin Instruments, Rockville, M, USA) bead beater apparatus (30 s at 5000 rpm, 2 times). After a pulse spin, lysate was transferred to a 1.5 mL protein LoBind tube (Eppendorf). The bead tube was washed with ¼ the lysis buffer volume, also transferred to the new tube. Samples were heated for 10 min at 80 °C while shaking at 1400 rpm. After cooling, the samples were spun for 10 min at 10,000× *g* to pellet insoluble material, and the supernatants were transferred to fresh tubes. Protein concentrations were determined using the bicinchoninic acid assay (BCA) (ThermoFisher Scientific) with bovine serum albumin (BSA) as the standard. 

### 2.4. SDS-PAGE

Volumes of 15 µg protein equivalent were diluted with reducing LDS (lithium dodecyl sulfate) sample buffer (5% β-mercaptoethanol) for SDS-PAGE, heated 5 min at 85 °C, loaded on an 18-well Criterion XT BisTris gel, and electrophoresed in MES running buffer (Bio-Rad, CA, USA). After fixation and staining with BioSafe stain (Bio-Rad), the sample lanes were divided into 6 equal vertical segments to fractionate the proteins by size prior to nano-flow liquid chromatography–electrospray tandem mass spectrometry (nanoLC–ESI–MS/MS) analysis. Segments were cut horizontally across all lanes using major protein bands common to all samples as guides. Each gel piece was cut into ~2mm pieces, transferred to PCR tubes, and stored in 200 mM Tris at 4 °C until trypsin digest.

### 2.5. Trypsin Digest

Proteins were destained in acetonitrile/50mM Tris pH 8.1 until clear, reduced with 50 mM TCEP/50 mM Tris pH 8.1 at 60 °C for 40 min, followed by alkylation using 25 mM iodoacetamide/50 mM Tris pH 8.1 at room temperature for 30 min in the dark. Proteins were in-gel digested with 0.16 µg trypsin (Promega Corporation, Madison, WI) in 25 mM Tris pH 8.1/0.0002% Zwittergent 3–16, at 37 °C overnight, followed by peptide extraction with 2% trifluoroacetic acid and acetonitrile. Extractions were dried and stored at -20 °C.

### 2.6. Label-Free Proteomics Acquisition: Nano-Flow Liquid Chromatography–Electrospray Tandem Mass Spectrometry (nanoLC–ESI–MS/MS) 

Tissue proteome was interrogated for label-free proteomics using an Ultimate 3000 RSLCnano HPLC system (Thermo Fisher Scientific) coupled to a Q-Exactive mass spectrometer (Thermo Fisher Scientific). Briefly, dried trypsin digested samples were suspended in 0.2% formic acid, 0.1% trifluoroacetic acid, and 0.002% zwittergent 3-16. The digest peptide mixture was loaded onto a 330 nL Halo 2.7 ES-C18 trap (Optimize Technologies, Oregon City, OR). Chromatography was performed using A solvent (98% water/2% acetonitrile/0.2% formic acid) and B solvent (80% acetonitrile/10% isopropanol/10% water/0.2 % formic acid), over a 2% to 45% B gradient for 90 min at 400 nL/min through the trap and a PicoFrit (New Objective, Woburn, MA) 100 mm × 33 cm column hand packed with Agilent Poroshell 120 EC C18 packing (Agilent, Santa Clara, CA). The Q-Exactive mass spectrometer was set to acquire MS1 survey scans from 340 to 1600 m/z at a resolution of 70,000 (200 m/z) with an automatic gain control (AGC) target of 3e6 ions and a maximum ion inject time of 60 ms. Survey scans were followed by HCD MS/MS scans on the top 15 ions at resolution 17,500 with an AGC target of 2e5 ions and a maximum ion inject time of 60 ms. Dynamic exclusion placed selected ions on an exclusion list for 40 s.

### 2.7. Data Analysis

MaxQuant software (version 1.5.1.2) [18], with its built-in Andromeda search engine (Max-Plank Institute) [19], was used to search the mass spectrometry generated data files. Peak features were time-aligned and searched against a human UniProt database of reviewed entries (release 2018_05) [20]. A minimum of two peptides was required for protein identification, with a single peptide being identified as unique and proteins identified with shared peptides are grouped together. An in-house script written in R programming language performed differential expression analysis using protein intensities. First, protein intensities of each sample were Log_2_ transformed and then quantile normalization was performed. For each protein, the normalized intensities observed in two groups of samples were compared using a Gaussian-linked generalized linear model. A Student’s *t*-test was used to detect the differentially expressed proteins between pairs of experimental groups. Proteins that differed between groups with a false discovery rate (FDR) corrected *p*-values (Benjamini–Hochberg–Yekutieli procedure) < 0.3, absolute Log_2_ fold change (FC) > 0.4, and no missing values in any of the samples were considered as statistically significant for this study. In order to include as many true positives as possible and to allow more accurate interpretations of the proteomics data due to the physiological interdependency between proteins [21], we accepted a higher-than-usual false positive rate. In addition, body mass index (BMI) was included as a covariate in the linear regression model. A post-hoc power analysis was performed using a web-based calculator developed by the Department of Bioinformatics and Computational Biology, MD Anderson Cancer Center, University of Texas (https://biostatistics.mdanderson.org/MicroarraySampleSize). 

### 2.8. Pathway Analysis

Pathway analysis was conducted using Ingenuity Pathway Analysis software (IPA; QIAGEN Inc., http://www.ingenuity.com/products/pathways_analysis.html, RRID: SCR_008653). IPA software compares the total amount of occurrences of these proteins in the provided dataset with their database by using computational algorithms. Proteins with their corresponding information of SwissProt/UnitProt identifier, Gaussian FDR, Gaussian *p*-values, and Log_2_FC were entered into IPA software. Then, IPA was instructed to analyze only proteins with an FDR < 0.3 and Log_2_FC >|0.4|. IPA employs Fisher’s exact test to detect significant enrichment. The content for this analysis was limited to humans and experimentally observed findings were used to determine the confidence level. The statistical significance level for the pathway analysis was *p* ≤ 0.05 as in a previous study [22]. 

### 2.9. Western Blots

Western blotting was used for validating the results of the pathway analysis. Frozen DLPCF samples were homogenized as previously described and the same protein lysates that were used for SDS-PAGE were used for Western blotting. An equal amount of protein (20 μg/well) was loaded onto 4%-20% polyacrylamide precast gels (Mini-PROTEAN TGX, Bio-Rad, CA, USA), and subjected to electrophoresis running at 100V constant for 90 min. Then proteins were transferred onto activated polyvinylidene fluoride membranes at 100V constant for 30 min. Membranes were blocked for 2 hours at room temperature with gentle shake in blocking buffer (5% BSA in TBST) and then probed overnight at 4 °C with primary polyclonal antibodies for potassium voltage-gated channel subfamily Q member 3 (KCNQ3) at 1:500 (Cat#19966-1-AP Proteintech, IL, USA) and gamma-aminobutyric acid type A receptor beta1 subunit (GABRB1) at 1:500 (Cat#AB9680, Millipore-Sigma, MA, USA) diluted in blocking buffer. Three 10-min washes in TBST were followed by incubation with horseradish peroxidase-conjugated secondary polyclonal antibody Anti-rabbit IgG (Cat#7074, Cell Signaling Technologies, MA, USA) 1:2000 dilution in 1% BSA in TBST for 1 h at room temperature. After three 10-min washes in TBST, membranes were developed by chemiluminescence reactions. Densitometric quantification of candidate proteins was performed using ChemiDoc MP (Bio-Rad) software. Band intensities were measured using Image Lab software v.3.0 (Bio-Rad). Expression levels were normalized to the levels of Glyceraldehyde 3-phosphate dehydrogenase (GAPDH) (1:4000; Cat#MAB374, Millipore-Sigma). Relative protein levels were compared between groups by one-tailed Student’s *t-*test and the *p*-values were corrected for multiple comparisons by the Bonferroni method.

## 3. Results

### 3.1. Demographics

No differences were observed between suicide and non-suicide cases for any of the tissue-related variables (postmortem interval and tissue pH). The non-suicide group presented higher BMI, the rest of the demographic and clinical variables (age, type of psychiatric disorder, last recorded mood, psychosis history, postmortem toxicology, and smoking status) did not present a difference between groups (Table 1).

### 3.2. Proteomics Analysis

**Figure 1:**
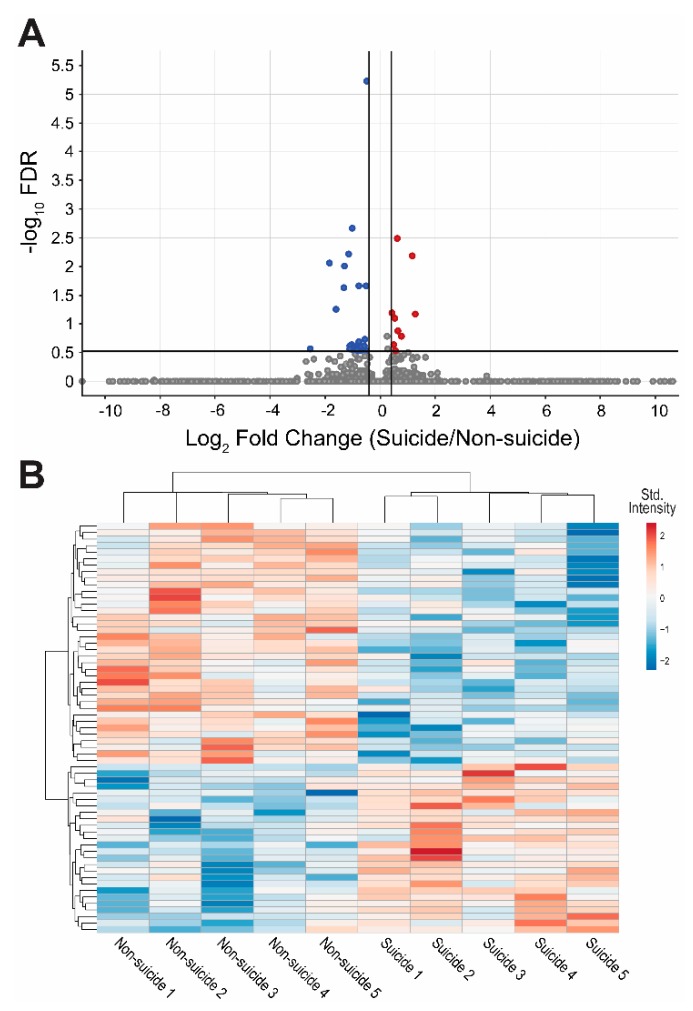
Proteomic profile identified by mass spectrometry-based untargeted proteomics. Proteins differentially expressed in the dorsolateral prefrontal cortex (DLPFC) of five individuals who died by suicide and five individuals who died by other causes (non-suicide) by having a false discovery rate (FDR) corrected *p*-values (Benjamini–Hochberg–Yekutieli procedure) < 0.3, absolute Log_2_ fold change (FC) > 0.4, and no missing values in any of the samples. (**A**) Volcano plot showing the downregulated proteins (blue), upregulated (red) and proteins that did not meet criteria (gray). (**B**) Heat map reflecting the expression values of proteins of interest from the non-suicide and suicide cases. Samples of each group are arranged in columns, proteins in rows. Blue shades show decreased expression and red shades show increased expression.

The label-free proteomic analysis detected 5162 proteins from the DLPFC tissues. The intensities of these unique proteins were compared between the suicide and non-suicide groups and 33 proteins were identified as significantly different with FDR < 0.3 and Log_2_FC >|0.4| (Table 2). Among these proteins, 24 of them were decreased and nine of them were increased in the suicide group (Figure 1). Furthermore, ten proteins had FDR < 0.05 among which eight were downregulated (Table 2). After adjusting the *p*-value for BMI, two out of the 33 presented proteins had an FDR < 0.3.

### 3.3. Pathway Analysis

**Figure 2:**
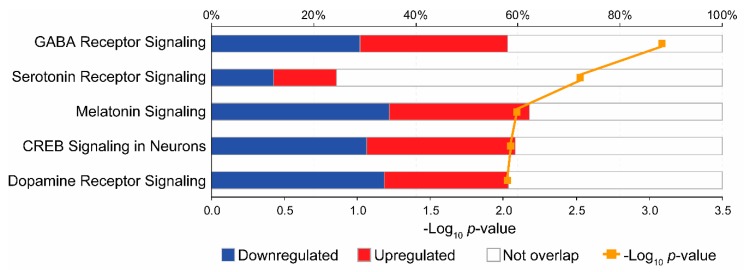
Top canonical enriched pathways identified by the Ingenuity Pathway Analysis software. This figure is based on proteins identified in the dataset of this study and their known associations across the pathways related to neurotransmitters and other nervous system signaling. The stacked bars indicate the percentage of proteins distributed according to regulation, i.e., blue (down), red (up), and open bars (no overlap with dataset) in each canonical pathway. The orange line represents the score for the likelihood that the proteins belonging to a specific canonical pathway, this is the “*p*-value of overlap” of the proteins in our dataset relative to the Ingenuity Pathway Analysis (IPA)’s predefined categories.

The top 33 proteins with an FDR < 0.3 and Log_2_FC >|0.4| were selected for pathway analysis by IPA and the described settings were applied. Five IPA canonical pathways were significantly enriched at *p* < 0.05 (Figure 2). These pathways were related to the following neurotransmitters and other nervous system signaling: GABA receptor signaling (*p* < 0.001), serotonin receptor signaling (*p* = 0.003), melatonin signaling (*p* = 0.008), CREB signaling in neurons (*p* = 0.009), and dopamine receptor signaling (*p* = 0.009). The three proteins that hit in the GABA receptor signaling pathway included KCNQ3 and GABRB1, both were downregulated, and ADCY5 was upregulated.

### 3.4. Validation of Top Protein Changes in DLPFC 

KCNQ3 and GABRB1 were selected for Western blot validation due to their involvement in the GABA receptor signaling pathway, which was the top significantly enriched pathway. This selection was reinforced by the relevance of these proteins in synapse functioning, this approach was similar to a previous study [23]. KCNQ3 levels were decreased in the DLPFC of individuals who died by suicide compared to individuals who died of non-suicide causes; nevertheless, these differences were not statistically significant after adjustment for multiple testing (Supplementary Figure S1). No significant group difference was detected in GABRB1 levels (Supplementary Figure S2). 

## 4. Discussion

In this study, mass spectrometry-based non-biased untargeted proteomics was used to identify the proteomic profile and canonical pathways that are associated with the biological mechanisms underlying suicide. Thirty-three protein levels showed a statistically significant difference in the DLPFC of individuals who died by suicide compared to individuals that died of other causes. Of these, KCNQ3 was the most significant hit and its downregulation was also observed as a trend in Western blot. The most notable finding in the pathway-level analysis was an enrichment of the GABA receptor signaling pathway. 

KCNQ3 (Potassium Voltage-Gated Channel Subfamily Q Member 3) encodes a protein that forms an M-channel that contributes to the native M current that regulates the subthreshold electrical excitability of neurons and responsiveness to synaptic input [24], and it can be directly activated by GABA [25]. Its role in brain electrical excitability is clinically highlighted by mutations of KCNQ-channel subunits that are involved in benign forms of familial epilepsy and intellectual disabilities [26]. There is also evidence supporting the possible role of M current dysregulation in patients with psychiatric disorders. The first line of evidence comes from genetic studies. The 6q14 locus, which was repeatedly shown to contain alleles increasing susceptibility to ADHD [27], schizophrenia [28], and bipolar disorder [29], was also shown to contain loss of function allele of KCNQ3 in affected family members with Benign Familial Neonatal Convulsions [30]. As M current is a result of the interaction between different KCNQ2/3 [24] and five [31] subunits, we might hypothesize that mutations in the 6q14 locus might exert their effects through dysregulation of M current, subsequent to dysfunction in channel subunits. The role of M current in neuronal excitability is another piece of supporting evidence. Particularly, KCNQ3 function and abnormalities have been associated with both MDD and BD. For instance, in a rodent model of resilience, mice that were unsusceptible to social defeat showed significant upregulation of KCNQ3 [32]. Supporting evidence of the role of KCNQ3 in resilience was found in a preclinical study that used a social defeat stress model of depression [33]. Gene therapy directed to elicit overexpression of KCNQ3 in dopaminergic neurons of the ventral tegmental area and the administration of ezogabine (retigabine) locally and systemically normalized depression-like behaviors and neural hyperactivity [33]. Ezogabine is an antiepileptic drug that acts primarily by opening neuronal voltage-gated potassium channels of the KCNQ (Kv7.2–7.5) family and it might also exert its therapeutic effects through the augmentation of currents mediated by GABA [34,35]. Recently, KCNQ-type potassium channels have been highlighted as a promising target for future drug discovery efforts in mood disorders, after an open-label study with ezogabine in patients with MDD showed an improvement in depressive and anhedonic symptoms [36]. Most of our individuals’ last reported mood was depression, the pathophysiological relevance of KCNQ3 in suicide could be the result of a similar abnormality from the resilience studies, where downregulation in the DLPFC of individuals who die by suicide, may derive in an unstable neuronal resting potential that may have an impact on resilience, depression, and suicide. 

The role of KCNQ2/3 has been studied in BD due to their interaction with ANK3, a widely recognize susceptibility gene for BD [37,38,39]. ANK3 protein helps to direct the localization of these potassium channels in the axon [38]. First, distinct splice variants of KCNQ3 interacting with KCNQ2 in BD patients were reported [40], while bioinformatics evidence supported significant interactions of KCNQ2 and KCNQ3 with ANK3 [38]. Following these findings, a study of postmortem prefrontal brain tissue of BD patients showed significant epigenetic changes of KCNQ3; the authors hypothesized that such changes may be involved in an ion channel dysfunction [41]. However, they could not determine if these results were due to the effects of mood-stabilizing agents [41]. It has also been reported that KCNQ2/3 potassium channels can be strongly activated by gabapentin [42]. Interestingly, a study showed the association between gabapentin use and a doubled risk of suicidality in patients with BD, compared to lithium [43]. Nevertheless, a population-based cohort study in Sweden clearly associated gabapentinoids, but not gabapentin, with an increased hazard of suicidal behavior [44]. Lithium has been shown in several meta-analyses to reduce suicidality [45,46,47]. One important mechanism of action for lithium is the blockade of GSK3β, which has many downstream targets including KCNQ2/3 [48]. Early pharmacological evidence suggests a potential mechanistic role of KCNQ3 regulators in mood disorders [36]. Our findings suggest that KCNQ3 effects should also be investigated in suicidal patients. 

Suicide phenotype is a broad spectrum to be analyzed and there is a well-known association between suicide and mental disorders. In our sample, all the individuals in both groups were diagnosed with major mood disorders, either MDD or BD. There is evidence that epigenetic alterations in the KCNQ3 gene may play a role in the etiopathogenesis of BD, as was suggested by lower DNA methylation in the postmortem prefrontal cortex that correlated with a significantly lower expression of KCNQ3 [41]. This finding was congruent with an earlier report of the association of BD with loci near KCNQ3 and ADCY8 [49]. Furthermore, some interactions between SNPs in association with bipolar disorder were described in a discovery GWAS dataset, of which the most significant ones were SNPs from KCNQ2 with SNPs from ANK3 [38]. 

GABRB1 is a subunit of the gamma-aminobutyric acid A receptor, which is a multi-subunit chloride channel that mediates the fastest inhibitory synaptic transmission in the central nervous system [50]. GABRB1 has an important role in the long-term consolidation of precise contextual memories and constrains generalized fear responses [51]. There is also evidence for the involvement of the GABRB1 gene in thalamus volume and interactive effects on intelligence [50]. GABRB1 protein levels were demonstrated to be altered specifically in the superior frontal cortex of subjects with autism [52], and in addictive behaviors, apparently, because it plays a role in the excitability of brain regions important in controlling reward-related behaviors [53]. GABA_A_ receptor mRNA expression has been shown to be altered in the depressed suicide brain, but there are no reports specifically regarding the B1 subunit [54]. In mass-spectrometry-based proteomics, we detected a reduction in GABRB1 protein levels in the DLPFC of suicide individuals, suggesting a possible alteration in the inhibitory synaptic transmission in this region. Nevertheless, we could not replicate this result by Western blot, which might be due to different reasons. Firstly, this could be a type II error due to our small sample size. Secondly, the threshold value for Log_2_FC may need to be more rigorous in order to appreciate the differences between groups. Lastly, it could be due to a difference between these techniques: mass-spectrometry-based detection in proteomics versus the epitope–antibody specific interaction in Western blot [55]. 

ADCY5 is a component of the GABA receptor pathway and has an important regulatory role catalyzing the generation of cyclic AMP (cAMP) from ATP [56] and showed higher levels in the DLPFC of the suicide completers. This protein contributes to corticostriatal plasticity and striatum-dependent learning [57]. ADCY5 has been implicated in several canonical pathways according to the predictions made by IPA software; in our list, it was present as well and enriched in pathways related to serotonin receptor signaling, dopamine receptor signaling, and CREB signaling in neurons. It would be pertinent to further explore the role of ADCY5 in future studies of suicide.

The key strength of this study is that the proteome of suicide completers was analyzed directly in brain tissue. However, some limitations should be noted. Because of the uncontrollable variability of individual human subjects, leading to increased *p*-values and FDRs, lower thresholds were considered to interrogate the data. Western blot validation was selectively performed in only two of the proteins of the most significant pathway; further studies are warranted to explore other relevant proteins. The small sample size led to a limited statistical power as our post-hoc power analysis revealed that a comparison of 5162 proteins for Log_2_FC < 0.4, with 0.4 standard deviations and at FDR < 0.3, 31 subjects per group would have been needed to achieve 80% power. It should be considered that suicide phenotype postmortem brain tissues are difficult to procure with an adequate postmortem interval that allows performing analyses of its proteins. Only males were included; hence, our study clearly underscores the need to use a larger sample size and analyze sex-specific differences. In addition, individuals could not be pair-matched perfectly for all known clinical and laboratory factors. Numerous clinical factors as medications, drugs of abuse, smoking, and BMI, just to mention a few, have a potential impact on the proteome and can certainly play a role as biological confounders. Nevertheless, apart from BMI, all of the aforementioned potential confounders were relatively matched between groups; therefore, we also included BMI as a covariate in the linear regression model (Table 2). In regard to medications, in our sample, we had three non-suicide individuals and three suicide individuals who were prescribed with medications during their last month of life (Supplemental Table S2), which made a balanced sample in relation to this factor. It is important to note here that this information has the proper limitations of any retrospective study. Therefore, the compliance of the prescribed medications cannot be completely known. In order to provide a wider picture of the medications, we also included information on the toxicology screen, and again it was well balance. There were two non-suicide individuals and two suicide individuals that presented a positive toxicology screening for medications (Supplemental Table S1). However, due to the variability of the half-life of these drugs, we cannot determine when exactly the medications were stopped. Interpretation of our results should consider that in our sample, all the individuals were diagnosed with a major mood disorder; further studies are needed to investigate if suicide represents a sign of the severity of mood disorders or if it can be considered as a separate entity. 

Here, we report potentially altered protein levels in the DLPFC of male suicide victims with mood disorders compared to individuals who died by other causes. There was a trend showing a reduction in protein levels of KCNQ3. In our sample, KCNQ3 contributed to the enrichment of the GABA receptor signaling pathway, the most significant canonical pathway identified. The suggested enrichment of the GABA pathway in suicide victims supports previous evidence regarding this neurotransmitter system alteration in this phenotype. These results in proteomics add to the increasing recent interest in KCNQ-type potassium channels as potential biological regulators in mood disorders, and suggest that their effects should also be investigated in suicidal individuals.

## Figures and Tables

**Table 1:** Subject demographics, clinical, and tissue-related features.

	Non-Suicide Death	Suicide	*p-*Value
Male (*n*)	5	5	-
Age (years; median and range) ^†^	52 (38–63)	56 (44–69)	0.529
Disorder (*n*; %) *			
Bipolar Disorder (Type I)	4 (80%)	3 (60%)	1.000
Major Depressive Disorder	1 (20%)	2 (40%)	
Last recorded mood (*n*; %) *			
Depression	4 (80%)	5 (100%)	1.000
Unknown	1 (20%)	0 (0%)	
Psychosis history (*n*; %) *			
Psychosis	3 (60%)	2 (60%)	1.000
No psychosis	2 (40%)	3 (40%)	
Postmortem toxicology (*n*; %) *			
Antidepressant medication	0	1 (20%)	1.000
Antipsychotic medication	1 (20%)	1 (20%)	1.000
Mood stabilizer medication	1 (20%)	0	1.000
Anxiolytic medication	1 (20%)	0	1.000
Alcohol	0	0	-
Cocaine	1 (20%)	0	1.000
Cannabinoids	0	0	-
Opiates	0	1 (20%)	1.000
Other drugs	2 (40%)	0	0.444
Body mass index (median and range) ^†^	49.4 (23–53.2)	23.8 (17.5–34.2)	0.047
Smoker (*n*; %) *^a^			
Yes (Current/Former)	3 (75%)	4 (80%)	1.000
No	1 (25%)	1 (20%)	
Postmortem interval (h; median and range) ^†^	22 (10–27.3)	20 (17–25)	1.000
pH (median and range) ^†^	6.08 (5.96–6.88)	6.69 (6.27– 7.02)	0.222

* Fisher’s exact test; ^†^ Mann–Whitney *U* test. ^a^ One individual with unknown smoking status

**Table 2:** Differentially expressed proteins in the dorsolateral prefrontal cortex between individuals who died by suicide and non-suicide death. These proteins presented a false discovery rate (FDR) < 0.3, no missing values in any of the samples, absolute Log_2_ fold change (FC) > 0.4, and were sorted by ascending FDR. Intensity makes reference to the signal intensity of detected ions as a function of the mass-to-charge ratio detected by nano-flow liquid chromatography–electrospray tandem mass spectrometry. * Log_2_FC (Suicide/Non-suicide). ** Student’s *t*-test. *** *p*-value and FDR after adjustment for body mass index (BMI) using a linear regression model.

Protein Name	Gene	Intensity		Log_2_ FC *	*p*-Value **	FDR	After BMI Adjustment	
		Non-suicide	Suicide				*p*-Value ***	FDR ***
Potassium voltage-gated channel subfamily Q member 3	KCNQ3	2.23 × 10^11^	1.6 × 10^11^	−0.481	2.10 × 10^−09^	5.93 × 10^−06^	0.005	0.463
Metallo-beta-lactamase domain-containing protein 1	MBLAC1	6.82 × 10^10^	3.38 × 10^10^	−1.011	1.16 × 10^−06^	0.002	0.028	0.790
Tripartite motif containing 36	TRIM36	9.18 × 10^11^	1.41 × 10^12^	0.614	2.31 × 10^−06^	0.003	0.004	0.418
RNA-binding motif protein X-linked	RBMX	3.74 × 10^12^	1.68 × 10^12^	−1.152	5.32 × 10^−06^	0.006	0.046	0.908
Adenylate cyclase 5	ADCY5	1.02 × 10^11^	2.3 × 10^11^	1.176	7.00 × 10^−06^	0.007	0.014	0.782
Ectonucleoside triphosphate diphosphohydrolase 2	ENTPD2	3.96 × 10^11^	1.11 × 10^11^	−1.834	1.09 × 10^−05^	0.009	0.034	0.827
NIMA related kinase 7	NEK7	1.1 × 10^11^	4.44 × 10^10^	−1.305	1.41 × 10^−05^	0.010	0.068	0.999
Sorting nexin 5	SNX5	4.16 × 10^12^	2.44 × 10^12^	−0.770	4.24 × 10^−05^	0.022	0.001	0.245
Fumarylacetoacetate hydrolase	FAH	1.09 × 10^12^	7.6 × 10^11^	−0.523	4.16 × 10^−05^	0.022	0.038	0.890
Megalencephalic leukoencephalopathy with subcortical cysts 1	MLC1	4.77 × 10^12^	1.92 × 10^12^	−1.315	5.00 × 10^−05^	0.023	0.044	0.893
Muscleblind-like splicing regulator 1	MBNL1	9.02 × 10^10^	2.97 × 10^10^	−1.601	1.27 × 10^−04^	0.055	0.127	0.999
Cytosolic carboxypeptidase	CBPC1	1.64 × 10^11^	2.22 × 10^11^	0.432	1.60 × 10^−04^	0.064	0.008	0.602
Hyperpolarization activated cyclic nucleotide gated potassium and sodium channel 2	HCN2	2.24 × 10^11^	5.45 × 10^11^	1.281	1.78 × 10^−04^	0.067	0.037	0.870
Phospholipase C-like 1 (inactive)	PLCL1	3.95 × 10^12^	5.69 × 10^12^	0.524	2.28 × 10^−04^	0.080	0.088	0.999
Dedicator of cytokinesis 1	DOCK1	4.24 × 10^11^	6.58 × 10^11^	0.635	4.01 × 10^−04^	0.133	0.059	0.965
Contactin 4	CNTN4	8.76 × 10^11^	1.5 × 10^12^	0.773	5.57 × 10^−04^	0.165	0.015	0.749
Connector enhancer of kinase suppressor of Ras 2	CNKSR2	1.41 × 10^12^	9.57 × 10^11^	−0.555	6.58 × 10^−04^	0.186	0.048	0.909
Calcium/calmodulin-dependent protein kinase type II subunit delta	CAMK2D	3.48 × 10^13^	2.03 × 10^13^	−0.777	7.60 × 10^−04^	0.204	0.043	0.906
Eukaryotic translation initiation factor 4E	EIF4E	2.8 × 10^12^	1.36 × 10^12^	−1.043	9.14 × 10^−04^	0.232	0.177	0.999
Ubiquitination factor E4A	UBE4A	2.5 × 10^12^	3.49 × 10^12^	0.485	9.44 × 10^−04^	0.232	0.010	0.680
Serine racemase	SRR	9.44 × 10^11^	4.41 × 10^11^	−1.096	0.001	0.240	0.114	0.999
Gamma-aminobutyric acid type A receptor beta1 subunit	GABRB1	7.94 × 10^11^	5.33 × 10^11^	−0.575	0.001	0.244	0.127	0.999
Membrane palmitoylated protein 2	MPP2	1.26 × 10^13^	7.09 × 10^12^	−0.835	0.001	0.244	0.060	0.970
Probable glutamate-tRNA ligase	EARS2	1.93 × 10^12^	1.04 × 10^12^	−0.893	0.001	0.263	0.026	0.776
RNA-binding protein EWS	EWSR1	6.18 × 10^11^	3.41 × 10^11^	−0.857	0.001	0.263	0.087	0.999
Sorting nexin-14	SNX14	2.76 × 10^10^	4.73 × 10^09^	−2.544	0.001	0.271	0.024	0.761
Arf-GAP with Rho-GAP domain, ANK repeat and PH domain-containing protein 1	ARAP1	7.04 × 10^09^	3.3 × 10^09^	−1.094	0.001	0.271	0.204	0.999
Amine oxidase (flavin-containing) A	MAOA	2.79 × 10^13^	1.84 × 10^13^	−0.603	0.002	0.277	0.190	0.999
Rho guanine nucleotide exchange factor 9	ARHGEF9	4.82 × 10^11^	2.39 × 10^11^	−1.012	0.002	0.282	0.061	0.975
Apoptosis-associated speck-like protein containing a CARD	PYCARD	8.75 × 10^10^	4.89 × 10^10^	−0.840	0.002	0.284	0.003	0.385
N-acetylserotonin O-methyltransferase-like protein	ASMTL	2.35 × 10^12^	1.41 × 10^12^	−0.739	0.002	0.292	0.191	0.999
Vesicle transport protein GOT1B	GOLT1B	9.75 × 10^10^	1.43 × 10^11^	0.556	0.002	0.295	0.001	0.235
Guanine nucleotide exchange C9orf72	C9orf72	4.16 × 10^11^	2.95 × 10^11^	-0.497	0.002	0.295	0.031	0.799

## Supplementary Materials

The following are available online at https://www.mdpi.com/2073-4425/11/3/256/s1, Figure S1: KCNQ3 protein levels were reduced in the dorsolateral prefrontal cortex of subjects who died by suicide compared to non-suicide subjects. Figure S2: Average GABRB1/GAPDH in the dorsolateral prefrontal cortex of five non-suicide cases and five suicide cases. Table S1: Toxicology screen and prescribed medications in the last month of life. Table S2: *Posthoc* Power Analysis and Sensitivity. Table S3: Thirty-three differentially expressed proteins between groups. File S1: Proteomics raw data.

## References

[1] Hedegaard H., Curtin S.C., Warner M. (2018). Suicide Mortality in the United States, 1999–2017. NCHS Data Brief.

[2] World Health Organization (2014). Preventing Suicide: A Global Imperative.

[3] Van Heeringen K., Mann J.J. (2014). The neurobiology of suicide. Lancet. Psychiatry.

[4] Benedetti F., Radaelli D., Poletti S., Locatelli C., Falini A., Colombo C., Smeraldi E. (2011). Opposite effects of suicidality and lithium on gray matter volumes in bipolar depression. J. Affect. Disord..

[5] Wagner G., Schultz C.C., Koch K., Schachtzabel C., Sauer H., Schlosser R.G. (2012). Prefrontal cortical thickness in depressed patients with high-risk for suicidal behavior. J. Psychiatr. Res..

[6] Rizk M.M., Rubin-Falcone H., Lin X., Keilp J.G., Miller J.M., Milak M.S., Sublette M.E., Oquendo M.A., Ogden R.T., Abdelfadeel N.A. (2018). Gray matter volumetric study of major depression and suicidal behavior. Psychiatry Res. Neuroimaging.

[7] Underwood M.D., Kassir S.A., Bakalian M.J., Galfalvy H., Mann J.J., Arango V. (2012). Neuron density and serotonin receptor binding in prefrontal cortex in suicide. Int. J. Neuropsychopharmacol..

[8] Kekesi K.A., Juhasz G., Simor A., Gulyassy P., Szego E.M., Hunyadi-Gulyas E., Darula Z., Medzihradszky K.F., Palkovits M., Penke B. (2012). Altered functional protein networks in the prefrontal cortex and amygdala of victims of suicide. PLoS ONE.

[9] Zhao J., Verwer R.W.H., van Wamelen D.J., Qi X.R., Gao S.F., Lucassen P.J., Swaab D.F. (2016). Prefrontal changes in the glutamate-glutamine cycle and neuronal/glial glutamate transporters in depression with and without suicide. J. Psychiatr. Res..

[10] Mann J.J., Currier D.M. (2010). Stress, genetics and epigenetic effects on the neurobiology of suicidal behavior and depression. Eur. Psychiatry J. Assoc. Eur. Psychiatr..

[11] Wilkins M.R., Sanchez J.C., Gooley A.A., Appel R.D., Humphery-Smith I., Hochstrasser D.F., Williams K.L. (1996). Progress with proteome projects: Why all proteins expressed by a genome should be identified and how to do it. Biotechnol. Genet. Eng. Rev..

[12] Martins-de-Souza D., Harris L.W., Guest P.C., Turck C.W., Bahn S. (2010). The role of proteomics in depression research. Eur. Arch. Psychiatry Clin. Neurosci..

[13] Turecki G. (2014). The molecular bases of the suicidal brain. Nat. Rev. Neurosci..

[14] Frye M.A., Nassan M., Jenkins G.D., Kung S., Veldic M., Palmer B.A., Feeder S.E., Tye S.J., Choi D.S., Biernacka J.M. (2015). Feasibility of investigating differential proteomic expression in depression: Implications for biomarker development in mood disorders. Transl. Psychiatry.

[15] Burger B., Hernandez Sanchez L.F., Lereim R.R., Barsnes H., Vaudel M. (2018). Analyzing the Structure of Pathways and Its Influence on the Interpretation of Biomedical Proteomics Data Sets. J. Proteome Res..

[16] Nussbeck S., Wemheuer W., Beier K. (2015). Why brain banking should be regarded as a special type of biobanking: Ethical, practical, and data-management challenges. J. Biorepository Sci. Appl. Med..

[17] Ho A.M., Winham S.J., Armasu S.M., Blacker C.J., Millischer V., Lavebratt C., Overholser J.C., Jurjus G.J., Dieter L., Mahajan G. (2019). Genome-wide DNA methylomic differences between dorsolateral prefrontal and temporal pole cortices of bipolar disorder. J. Psychiatr. Res..

[18] Cox J., Mann M. (2008). MaxQuant enables high peptide identification rates, individualized p.p.b.-range mass accuracies and proteome-wide protein quantification. Nat. Biotechnol..

[19] Cox J., Neuhauser N., Michalski A., Scheltema R.A., Olsen J.V., Mann M. (2011). Andromeda: A peptide search engine integrated into the MaxQuant environment. J. Proteome Res..

[20] Consortium T.U. (2018). UniProt: A worldwide hub of protein knowledge. Nucleic Acids Res..

[21] Martins-de-Souza D., Guest P.C., Harris L.W., Vanattou-Saifoudine N., Webster M.J., Rahmoune H., Bahn S. (2012). Identification of proteomic signatures associated with depression and psychotic depression in post-mortem brains from major depression patients. Transl. Psychiatry.

[22] Oliveros A., Starski P., Lindberg D., Choi S., Heppelmann C.J., Dasari S., Choi D.S. (2017). Label-Free Neuroproteomics of the Hippocampal-Accumbal Circuit Reveals Deficits in Neurotransmitter and Neuropeptide Signaling in Mice Lacking Ethanol-Sensitive Adenosine Transporter. J. Proteome Res..

[23] Pinacho R., Villalmanzo N., Meana J.J., Ferrer I., Berengueras A., Haro J.M., Villen J., Ramos B. (2016). Altered CSNK1E, FABP4 and NEFH protein levels in the dorsolateral prefrontal cortex in schizophrenia. Schizophr. Res..

[24] Wang H.S., Pan Z., Shi W., Brown B.S., Wymore R.S., Cohen I.S., Dixon J.E., McKinnon D. (1998). KCNQ2 and KCNQ3 potassium channel subunits: Molecular correlates of the M-channel. Science.

[25] Manville R.W., Papanikolaou M., Abbott G.W. (2018). Direct neurotransmitter activation of voltage-gated potassium channels. Nat. Commun.

[26] Okada M., Zhu G., Hirose S., Ito K.I., Murakami T., Wakui M., Kaneko S. (2003). Age-dependent modulation of hippocampal excitability by KCNQ-channels. Epilepsy Res..

[27] Ogdie M.N., Fisher S.E., Yang M., Ishii J., Francks C., Loo S.K., Cantor R.M., McCracken J.T., McGough J.J., Smalley S.L. (2004). Attention deficit hyperactivity disorder: Fine mapping supports linkage to 5p13, 6q12, 16p13, and 17p11. Am. J. Hum. Genet..

[28] Martinez M., Goldin L.R., Cao Q., Zhang J., Sanders A.R., Nancarrow D.J., Taylor J.M., Levinson D.F., Kirby A., Crowe R.R. (1999). Follow-up study on a susceptibility locus for schizophrenia on chromosome 6q. Am. J. Med. Genet..

[29] Dick D.M., Foroud T., Flury L., Bowman E.S., Miller M.J., Rau N.L., Moe P.R., Samavedy N., El-Mallakh R., Manji H. (2003). Genomewide linkage analyses of bipolar disorder: A new sample of 250 pedigrees from the National Institute of Mental Health Genetics Initiative. Am. J. Hum. Genet..

[30] Gargus J.J. (2006). Ion channel functional candidate genes in multigenic neuropsychiatric disease. Biol. Psychiatry.

[31] Mooney D.M., Zhang L., Basile C., Senatorov V.V., Ngsee J., Omar A., Hu B. (2004). Distinct forms of cholinergic modulation in parallel thalamic sensory pathways. Proc. Natl. Acad. Sci. USA.

[32] Krishnan V., Han M.H., Graham D.L., Berton O., Renthal W., Russo S.J., Laplant Q., Graham A., Lutter M., Lagace D.C. (2007). Molecular adaptations underlying susceptibility and resistance to social defeat in brain reward regions. Cell.

[33] Friedman A.K., Juarez B., Ku S.M., Zhang H., Calizo R.C., Walsh J.J., Chaudhury D., Zhang S., Hawkins A., Dietz D.M. (2016). KCNQ channel openers reverse depressive symptoms via an active resilience mechanism. Nat. Commun.

[34] Stafstrom C.E., Grippon S., Kirkpatrick P. (2011). Ezogabine (retigabine). Nat. Rev. Drug Discov..

[35] Cao Y., Bartolomé-Martín D., Rotem N., Rozas C., Dellal S.S., Chacon M.A., Kadriu B., Gulinello M., Khodakhah K., Faber D.S. (2015). Rescue of homeostatic regulation of striatal excitability and locomotor activity in a mouse model of Huntington’s disease. Proc. Natl. Acad. Sci. USA.

[36] Tan A., Costi S., Morris L.S., Van Dam N.T., Kautz M., Whitton A.E., Friedman A.K., Collins K.A., Ahle G., Chadha N. (2018). Effects of the KCNQ channel opener ezogabine on functional connectivity of the ventral striatum and clinical symptoms in patients with major depressive disorder. Mol. Psychiatry.

[37] Lopez A.Y., Wang X., Xu M., Maheshwari A., Curry D., Lam S., Adesina A.M., Noebels J.L., Sun Q.Q., Cooper E.C. (2017). Ankyrin-G isoform imbalance and interneuronopathy link epilepsy and bipolar disorder. Mol. Psychiatry.

[38] Judy J.T., Seifuddin F., Pirooznia M., Mahon P.B., Jancic D., Goes F.S., Schulze T., Cichon S., Noethen M., Rietschel M. (2013). Converging Evidence for Epistasis between ANK3 and Potassium Channel Gene KCNQ2 in Bipolar Disorder. Front. Genet..

[39] Judy J.T., Zandi P.P. (2013). A review of potassium channels in bipolar disorder. Front. Genet..

[40] Borsotto M., Cavarec L., Bouillot M., Romey G., Macciardi F., Delaye A., Nasroune M., Bastucci M., Sambucy J.L., Luan J.J. (2007). PP2A-Bgamma subunit and KCNQ2 K+ channels in bipolar disorder. Pharm. J..

[41] Kaminsky Z., Jones I., Verma R., Saleh L., Trivedi H., Guintivano J., Akman R., Zandi P., Lee R.S., Potash J.B. (2015). DNA methylation and expression of KCNQ3 in bipolar disorder. Bipolar Disord..

[42] Manville R.W., Abbott G.W. (2018). Gabapentin Is a Potent Activator of KCNQ3 and KCNQ5 Potassium Channels. Mol. Pharmacol..

[43] Leith W.M., Lambert W.E., Boehnlein J.K., Freeman M.D. (2019). The association between gabapentin and suicidality in bipolar patients. Int. Clin. Psychopharmacol..

[44] Molero Y., Larsson H., D’Onofrio B.M., Sharp D.J., Fazel S. (2019). Associations between gabapentinoids and suicidal behaviour, unintentional overdoses, injuries, road traffic incidents, and violent crime: Population based cohort study in Sweden. BMJ.

[45] Smith K.A., Cipriani A. (2017). Lithium and suicide in mood disorders: Updated meta-review of the scientific literature. Bipolar Disord..

[46] Roberts E., Cipriani A., Geddes J.R., Nierenberg A.A., Young A.H. (2017). The evidence for lithium in suicide prevention. Br. J. Psychiatry.

[47] D’Anci K.E., Uhl S., Giradi G., Martin C. (2019). Treatments for the Prevention and Management of Suicide: A Systematic Review. Ann. Intern. Med..

[48] Wildburger N.C., Laezza F. (2012). Control of neuronal ion channel function by glycogen synthase kinase-3: New prospective for an old kinase. Front. Mol. Neurosci..

[49] Zhang P., Xiang N., Chen Y., Sliwerska E., McInnis M.G., Burmeister M., Zollner S. (2010). Family-based association analysis to finemap bipolar linkage peak on chromosome 8q24 using 2,500 genotyped SNPs and 15,000 imputed SNPs. Bipolar Disord..

[50] Zhu B., Chen C., Xue G., Lei X., Li J., Moyzis R.K., Dong Q., Lin C. (2014). The GABRB1 gene is associated with thalamus volume and modulates the association between thalamus volume and intelligence. Neuroimage.

[51] Lynch J.F., Winiecki P., Gilman T.L., Adkins J.M., Jasnow A.M. (2017). Hippocampal GABAB(1a) Receptors Constrain Generalized Contextual Fear. Neuropsychopharmacology.

[52] Fatemi S.H., Reutiman T.J., Folsom T.D., Rooney R.J., Patel D.H., Thuras P.D. (2010). mRNA and protein levels for GABAAalpha4, alpha5, beta1 and GABABR1 receptors are altered in brains from subjects with autism. J. Autism Dev. Disord..

[53] Duka T., Nikolaou K., King S.L., Banaschewski T., Bokde A.L., Buchel C., Carvalho F.M., Conrod P.J., Flor H., Gallinat J. (2017). GABRB1 Single Nucleotide Polymorphism Associated with Altered Brain Responses (but not Performance) during Measures of Impulsivity and Reward Sensitivity in Human Adolescents. Front. Behav. Neurosci..

[54] Poulter M.O., Du L., Zhurov V., Palkovits M., Faludi G., Merali Z., Anisman H. (2010). Altered Organization of GABA(A) Receptor mRNA Expression in the Depressed Suicide Brain. Front. Mol. Neurosci..

[55] Martins-de-Souza D., Guest P.C., Vanattou-Saifoudine N., Harris L.W., Bahn S. (2011). Proteomic technologies for biomarker studies in psychiatry: Advances and needs. Int. Rev. Neurobiol..

[56] Vatner S.F., Park M., Yan L., Lee G.J., Lai L., Iwatsubo K., Ishikawa Y., Pessin J., Vatner D.E. (2013). Adenylyl cyclase type 5 in cardiac disease, metabolism, and aging. Am. J. Physiol. Heart Circ. Physiol..

[57] Kheirbek M.A., Britt J.P., Beeler J.A., Ishikawa Y., McGehee D.S., Zhuang X. (2009). Adenylyl cyclase type 5 contributes to corticostriatal plasticity and striatum-dependent learning. J. Neurosci..

